# Plant Factories Are Heating Up: Hunting for the Best Combination of Light Intensity, Air Temperature and Root-Zone Temperature in Lettuce Production

**DOI:** 10.3389/fpls.2020.592171

**Published:** 2021-01-28

**Authors:** Laura Carotti, Luuk Graamans, Federico Puksic, Michele Butturini, Esther Meinen, Ep Heuvelink, Cecilia Stanghellini

**Affiliations:** ^1^Department of Biological, Geological and Environmental Sciences, Alma Mater Studiorum, University of Bologna, Bologna, Italy; ^2^Greenhouse Horticulture, Wageningen University and Research, Wageningen, Netherlands; ^3^Horticulture and Product Physiology, Wageningen University and Research, Wageningen, Netherlands

**Keywords:** climate management, dry matter allocation, efficiency, leaf expansion, production climate, resource use efficiency, vertical farm, light use efficiency

## Abstract

This study analyzed interactions among photon flux density (PPFD), air temperature, root-zone temperature for growth of lettuce with non-limiting water, nutrient, and CO_2_ concentration. We measured growth parameters in 48 combinations of a PPFD of 200, 400, and 750 μmol m^–2^ s^–1^ (16 h daylength), with air and root-zone temperatures of 20, 24, 28, and 32°C. Lettuce (*Lactuca sativa cv*. Batavia Othilie) was grown for four cycles (29 days after transplanting). Eight combinations with low root-zone (20 and 24°C), high air temperature (28 and 32°C) and high PPFD (400 and 750 μmol m^–2^ s^–1^) resulted in an excessive incidence of tip-burn and were not included in further analysis. Dry mass increased with increasing photon flux to a PPFD of 750 μmol m^–2^ s^–1^. The photon conversion efficiency (both dry and fresh weight) decreased with increasing photon flux: 29, 27, and 21 g FW shoot and 1.01, 0.87, and 0.76 g DW shoot per mol incident light at 200, 400, and 750 μmol m^–2^ s^–1^, respectively, averaged over all temperature combinations, following a concurrent decrease in specific leaf area (SLA). The highest efficiency was achieved at 200 μmol m^–2^ s^–1^, 24°C air temperature and 28°C root-zone temperature: 44 g FW and 1.23 g DW per mol incident light. The effect of air temperature on fresh yield was linked to all leaf expansion processes. SLA, shoot mass allocation and water content of leaves showed the same trend for air temperature with a maximum around 24°C. The effect of root temperature was less prominent with an optimum around 28°C in nearly all conditions. With this combination of temperatures, market size (fresh weight shoot = 250 g) was achieved in 26, 20, and 18 days, at 200, 400, and 750 μmol m^–2^ s^–1^, respectively, with a corresponding shoot dry matter content of 2.6, 3.8, and 4.2%. In conclusion, three factors determine the “optimal” PPFD: capital and operational costs of light intensity vs the value of reducing cropping time, and the market value of higher dry matter contents.

## Introduction

Recent social developments have increased the allure of locally produced food and urban horticulture is increasingly seen as an option to produce locally (Benke and Tomkins, 2017; Shamshiri et al., 2018). However, an economically viable exploitation of expensive urban land for agriculture is only possible for high-value, high-yield crops. Plant factories, also known as vertical farms, are capable of cultivating crops on multiple layers and achieving high crop productivity and uniformity, without any need for crop protection chemicals (Graamans et al., 2018; SharathKumar et al., 2020). Such production systems are completely insulated from the exterior climate and control light (spectrum, intensity, and photoperiod), temperature, relative humidity, and CO_2_ concentration. They are typically used to produce small, “stackable” plants with a short production cycle, such as leafy vegetables and herbs, seedlings and high-value medicinal crops (Kozai, 2013). Production costs in plant factories are higher than in any other agricultural system relying on sunlight, with estimates by the Rabobank (Van Rijswick, 2018) projecting at least twice the production cost in comparison with the nearest competitor: the high-tech, heated glasshouse. As the energy requirement for climatization (lighting, cooling, and dehumidification) is a major component of the production costs, climate management should be optimized to balance marginal yield, and marginal energy requirement. Systems with full climate control, such as plant factories, allow for the optimization of the production climate when the crop response to different climate factors is known.

The response of plant development and growth to environmental conditions, known as phenotypic plasticity, is species-specific (Sultan, 2003). Light intensity, CO_2_ concentration and, to a lesser extent, temperature are the main environmental factors that determine photosynthesis and therefore crop growth and production. The ability of leaves to intercept light is determined by the leaf area, orientation and optical properties (Héraut-Bron et al., 2001). Plants have evolved different mechanisms to adapt to the light environment. For instance, plants grown in low light maximize light interception by partitioning a high proportion of assimilates toward the leaves (Shoot Mass Fraction, SMF, Poorter and Nagel, 2000) and by increasing their specific leaf area (SLA, leaf area per unit dry matter; Fan et al., 2013). Leaf area extension consists of two components: an increase in volume (by cell expansion) and an increase in dry matter, also known as structural growth (by leaf initiation and cell multiplication; Pantin et al., 2011).

Crop photosynthesis does not depend much on temperature, provided it is within a “reasonable range” (Körner et al., 2009). High temperature stress can induce changes in, e.g., water relations, osmolyte accumulation, photosynthetic activity, hormone production, and cell membrane thermostability (Waraich et al., 2012). Furthermore, temperature directly determines the rate of development of new organs in a species-specific way (Hatfield and Prueger, 2015). This influences marketable yield, which is determined by the amount of dry matter and the water content of the harvestable product. The amount of dry matter is determined by the dry matter production (net assimilation) and its allocation among organs (sinks; e.g., Marcelis et al., 1998). A reduced water content of the harvestable product is often an indicator of better quality (e.g., Acharya et al., 2017). The role of temperature in the aforementioned processes has been investigated in greater depth for leaf and air temperature than for root-zone temperature. Nonetheless, for lettuce there are indications that cooling the root zone may have a mitigating effect under high air temperatures (Thompson et al., 1998; He et al., 2001) and that the optimal root-zone temperature may increase with light intensity (Gosselin and Trudel, 1984; Frantz et al., 2004).

**FIGURE 1:**
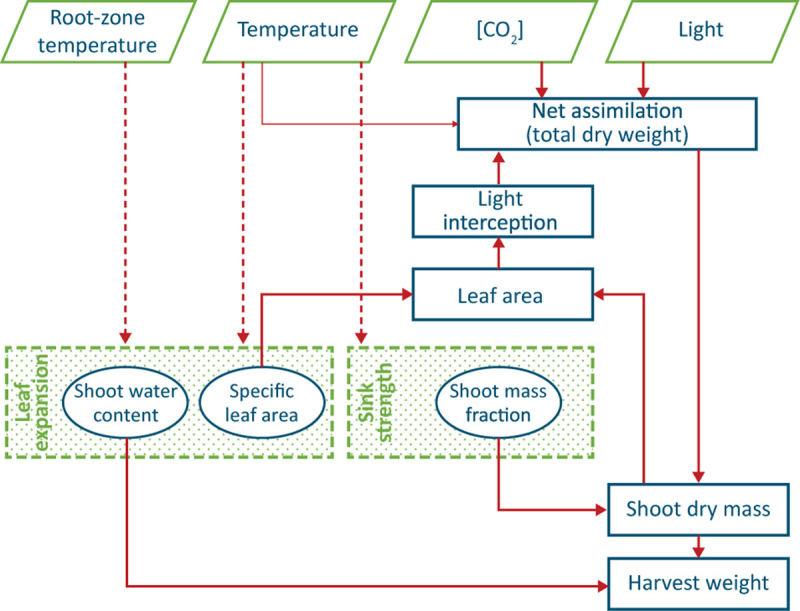
Conceptual model of production of a leafy vegetable, such as lettuce. Thick arrows indicate well-known causal relationships, thin arrows a weak relationship and dashed arrows circumstantial evidence. The two shaded processes are not yet fully understood, and the three encircled ‘entities’ are often regarded as constant values. Water and nutrient supply are assumed not to be limiting and thus do not appear in the scheme.

The lack of obvious conclusions above is probably the reason for most existing models of leafy crops (such as Van Henten, 1994) to have a SMF, SLA and shoot water content as parameters, and furthermore to not take into account possible effects of root zone temperature on crop growth (Figure 1).

Understanding the relationship between light, air temperature and root-zone temperature and lettuce growth allows for the optimization of the growing conditions for the plants. This optimization is particularly interesting for systems with extensive climate control, such as plant factories. Such closed systems need cooling whenever light is supplied, whereas maintaining a high CO_2_ concentration is relatively cheap. Therefore, it makes economic sense to explore yield response to climate conditions that are not typical of more conventional growing environments (including heated greenhouses with natural ventilation) where there is always a high correlation among factors such as solar radiation, air and root-zone temperature. Therefore the objective of this paper is to extend our knowledge about plant processes, such as leaf expansion and dry matter allocation, in order to determine whether and how they could be manipulated through climate management. In view of the extended climate manipulation options in a vertical farm, we have also considered “unnatural” combinations of light intensity and root zone and ambient temperature independently. Our assumption was that production increases with light intensity, as long as other factors are not limiting. We also expected a higher optimal temperature at higher light intensity to maintain a balance between source and sink strength (Gent and Seginer, 2012).

## Materials and Methods

### Growth Conditions, Treatments and Analysis

**FIGURE 2:**
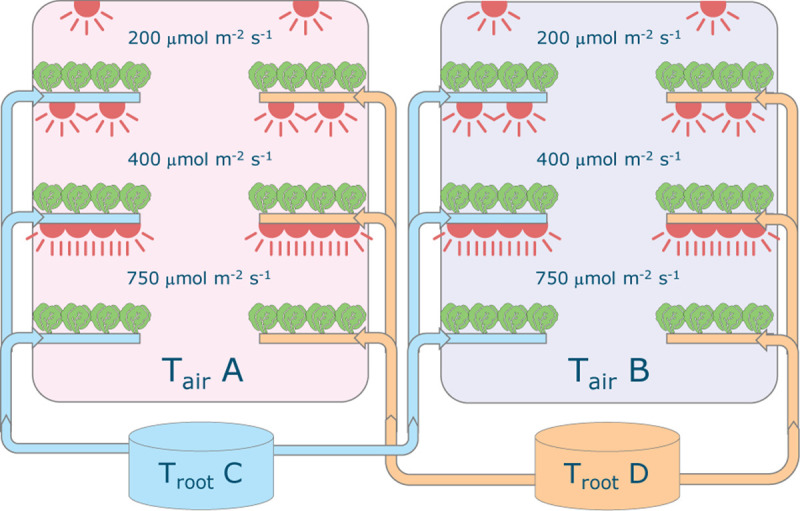
Schematic representation of the experimental set-up. Each of the two climate rooms had three light intensity levels installed (200, 400 and 750 μmol m^–2^ s^–1^) and featured two root-zone temperatures. All combinations of 20, 24, 28, 32°C for air and root-zone temperatures were tested in four successive crop cycles. The lights were on-off for 16–8 hours, respectively and temperature was maintained constant. Carbon dioxide concentration was 1200 μmol mol^–1^ throughout.

Plants of *Lactuca sativa* cv. Batavia Othilie were grown in two climate rooms at Wageningen University & Research (Netherlands) in a hydroponic (deep water culture) system (Figure 2) with different combinations of air temperature (20, 24, 28, and 32°C), root-zone temperature (20, 24, 28, and 32°C) and light intensity (200, 400, and 750 μmol m^–2^ s^–1^). Four sequential growth cycles were conducted from December 2018 to May 2019, for a total of 48 treatments. The photoperiod was 16/8 h (day/night) throughout the entire cycle and CO_2_ concentration was kept constant at 1,200 μmol mol^–1^. Air was continuously circulated, resulting in an air exchange rate of approximately 40 times per hour. The relative humidity was adjusted based on the temperature, to keep similar vapor pressure deficit among the various treatments (about 5.8 and 3.4 hPa, day and night, respectively, that is a higher relative humidity in the dark period).

Lettuce seeds were sown in stonewool cubes (4 × 4 cm; Rockwool Grodan, Roermond, Netherlands) and covered with plastic (dark and at 18°C) in a separate, germination room. After 2 to 3 days, the seeds germinated and the plastic was removed. Temperature was maintained at 20°C, vapor pressure deficit 5.8 and 3.5 hPa during light and dark period, respectively, and a photosynthetic photon flux density (PPFD) of 200 μmol m^–2^ s^–1^ (photoperiod 16 h) was provided by fluorescent tubes. The temperature of the germination room was increased gradually over the course of 3 days to acclimate the plants to the air temperatures of 28 and 32°C. In all cases uniform lettuce seedlings were selected after 19 days before being transplanted at random into the floaters of the deep water culture system (each floater 20 × 80 cm, 4 plants). After transplanting, the air temperature and root zone temperatures were gradually increased over the course of 48 h to reach the final temperatures for the treatment 32°C. The treatment with the highest light intensity (750 μmol m^–2^ s^–1^) was shaded for 48 h to allow the plants to acclimate to the light levels.

Each climate cell contained six production layers, three on the left side and three on the right side (Figure 2). On each layer a deep flow tank (8 cm deep) contained 15 floating trays of 4 plants each, resulting in a density of 25 plants m^–2^. The nutrient solution had an electronic conductivity (EC) of 2.0 dS m^–1^ and was composed of the following ions in mmol L^–1^: 12 NO_3_^–^, 1 NH_4_^+^, 6 K^+^, 3 Ca^2+^, 0.84 Mg^2+^, 1.1 H_2_PO_4_^–^, 0.79 SO_4_^2–^, and in μmol L^–1^: 50 Fe, 8 Mn, 5 Zn, 40 B, 0.5 Cu, and 0.5 Mo. A new solution using the same recipe was prepared for each cycle and any refill. EC and pH were checked weekly and the actual concentration was measured halfway through the each cycle to check whether corrections were needed^1^. The dissolved oxygen was maintained at saturation with a water oxygenator and root-zone temperature set-point was maintained using a heat exchanger in each of the two nutrient solution tanks. The tanks were placed outside of the climate rooms to exclude heat exchange.

The climate rooms were thermally insulated and the air temperature, humidity levels and CO_2_ concentration was managed per room. The temperature of the nutrient solution for the root zone was varied per side of the climate room and the light intensity was varied per production layer. Four air and root zone temperatures (20, 24, 28, or 32°C) and three light intensities (200, 400, or 750 μmol m^–2^ s^–1^, corresponding to Daily Light Integrals of 11.5, 23.0, and 43.2 mol m^–2^ d^–1^, respectively) were used during the experiment. Each production layer, characterized by a specific combination of air temperature, root zone temperature and light intensity, corresponded to a treatment.

Air temperature, root-zone temperature and relative humidity were continuously monitored from the day of transplanting (19 DAS). Each climate room was provided with 6 ventilated sensors, one for each layer (Sensirion SHT75, WiSensys, Wireless Value, Netherlands) measuring air temperature (±0.3°C) and relative humidity (±1.8%) and with 4 sensors (two for each side, top and bottom layer, SHT71, WiSensys, Wireless Value, Netherlands) for the root-zone temperature (±0.3°C). CO_2_ was measured and controlled using the central climate control box. Measurements were recorded at 5 min intervals. PPFD was provided with two different types of LED modules: For the 200 μmol m^–2^ s^–1^ treatment the Philips GP LED production module (2.2 DR/W 150 cm LB HO) and for the 400 and 750 μmol m^–2^ s^–1^ treatments the Philips GP LED Toplight (1.2 DR/W LB 400V) were used. The application of different lighting modules was necessary to ensure the required light intensities, as well as an adequate spatial distribution. These factors were considered to be more consequential for the experiment than the resulting minor difference in spectral distribution (see Supplementary Figure 1). Light intensity was measured using a quantum sensor (LI-190) at the start of the cycle, on 36 spots on each layer, at the height of the top of transplanted plants. Light spectrum was measured using a spectroradiometer (Jeti specbos 1211).

### Crop Measurements and Statistical Analysis

**FIGURE 3:**
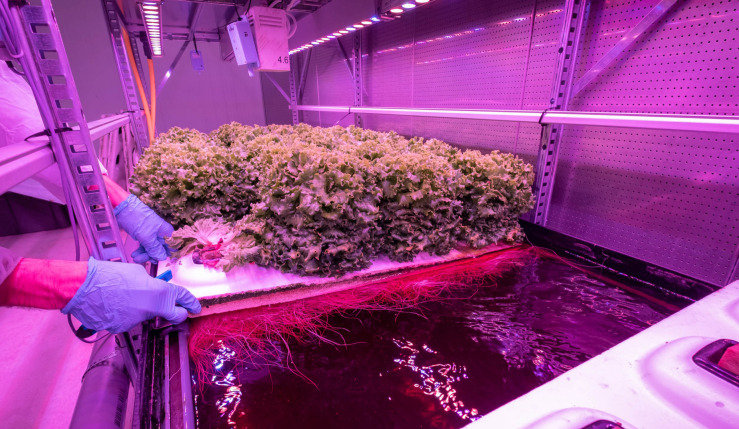
One of the final harvests in the 750 μmol m^–2^ s^–1^ treatment.

Each crop cycle lasted 29 days after transplanting (DAT), which gave heads of market size (250 g) in the 200 μmol m^–2^ s^–1^ treatments. Destructive harvests for determining leaf, stem and root fresh and dry weight (ventilated oven, 24 h at 70°C followed by 24 h at 105°C) and leaf area per plant (LI-31000C, LI-COR Biosciences, United States) were conducted twice a week, for a total of nine harvests. Tip-burn occurrence (% of plants affected to any extent) was evaluated each time but no severity scale was used. The external 3 floaters at each side of a layer were considered as border floaters. The central 9 floaters contained the experimental plants for each layer. The central floater was extracted each time and the four plants (replicas) were destructively measured (Figure 3). The remaining floaters were slid to the center to ensure uniformity and maintain a continuous canopy and density.

Eight combinations (*T*_air_ ≥ 28°C, *T*_root_ ≤ 24°C, and PPFD ≥ 400 μmol m^–2^ s^–1^) resulted in excessive (>50%) incidence of tip-burn from DAT 6 and were therefore excluded from further analysis. Data of 4 replicate plants were averaged and represent one experimental unit. An Analysis of Variance (ANOVA; SPSS 26th edition) was conducted on final total dry weight, shoot/root ratio, SLA, and light use efficiency, treating the data from the four experiments as a 3-way full-factorial incomplete randomized design. Sources of variance were the main effects of light intensity, air temperature and root-zone temperature and their 2-way interactions whereas the 3-way interaction was taken as a residual term, because each combination of a light intensity, a root temperature and an air temperature was conducted only once.

## Results

### Treatments

The realized climate conditions were maintained within 3% (average temperature) and 6% (light intensity) of the desired setpoints (Table 1) and the standard deviation was never more than 5% (air temperature); 2% (root temperature); and 6% (spatial distribution of light intensity).

**TABLE 1:** Average ± standard deviation of the measured temperature and light levels for each setpoint.

Set-point (°C)	20	24	28	32
Measured air temperature (°C)	19.3 ± 1.0	23.6 ± 0.8	28.6 ± 1.4	32.6 ± 1.3
Measured root temperature (°C)	20.2 ± 0.2	24.1 ± 0.4	27.8 ± 0.6	31.4 ± 0.2
Set-point PPFD (μmol m^–2^ s^–1^)	200	400	750	
Measured PPFD (μmol m^–2^ s^–1^)	197 ± 8	425 ± 24	741 ± 24	

*The listed measured air temperature is the mean of six sensors per climate room. The listed root-zone temperature is the mean of four sensors per climate room, two per side. Temperature data was recorded at an interval of 5 min and was averaged over the full growth cycle, during each experiment. Light intensity was measured at 36 spots on each layer, at crop height, before the cycles.*

The limited nature of tip-burn observations (fraction of plants affected) did not allow for a detailed analysis, but indicated that vapor condensation on the growing tip was the most likely cause in the combinations that had to be discarded. In all cases, tip-burn occurrence increased with air temperature.

### Yield

**FIGURE 4:**
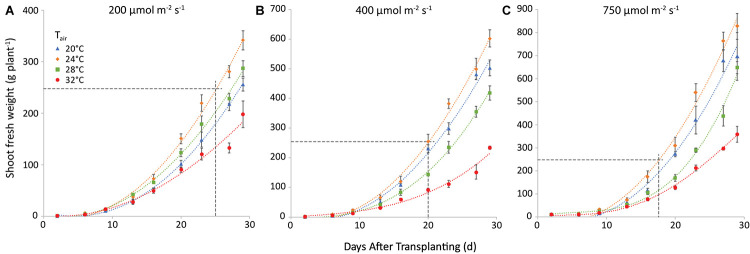
Shoot fresh weight (g plant^−1^) at a root temperature of 28°C, at light intensities of 200 **(A)**, 400 **(B)** and 750 **(C)** μmol m^−2^ s^−1^ and at air temperatures as indicated. The points represent the values at the different harvests (days after transplanting) and are the average of four plants, shown with the standard error of the mean. Note that the *y*-axis scale differs among the 3 panels, the horizonal line gives in each case the market size of 250 g. The curves are polynomials to increase legibility.

Yield increased with light intensity as expected, and there was an obvious effect of air temperature, as 24°C resulted in the highest yield and 32°C the lowest at all light intensities. The effect of root temperature (not shown) was smaller in all cases and less uniform. Figure 4 shows the combined effect of air temperature and light intensity on fresh weight of shoot, at a root zone temperature of 28°C (the one that warranted the highest weight in most cases). Raising light intensity from 200 to 400 μmol m^–2^ s^–1^ could shorten the time needed to reach market weight by 8 days at an air temperature of 20°C, 5 days at 24°C, and 3 at 28°C. Raising it further, to 750 μmol m^–2^ s^–1^ would shave off only another 2 days in all cases.

**FIGURE 5:**
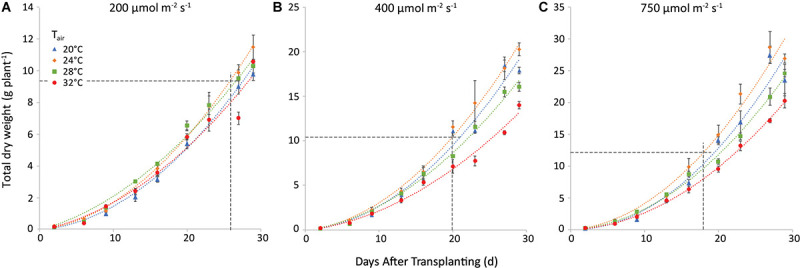
Total plant dry weight (g plant^–1^) at a root-zone temperature of 28 C and light intensities of 200 **(A)**, 400 **(B)** and 750 **(C)** μmol m^−2^ s^−1^. The four air temperatures are represented by different symbols/colors. The data points represent the values at the different harvests (days after transplanting) and are the average of four plants, shown with the standard error of the mean. The dashed lines represent the day when shoot market yield was reached (see Figure 4) and the corresponding total dry weight. Note that the *y*-axis scale differs among the 3 panels. The curves are polynomials to increase legibility.

The trend of total plant dry weight (TDW) was similar. Nevertheless, both air and root zone temperatures influenced TDW. The effect of air temperature is illustrated in Figure 5 for a root-zone temperature of 28°C. At equal air and root-zone temperature (not shown), the highest final dry weights were observed at 24°C (10.0, 16.3, and 28.1 g plant^–1^ at 200, 400, and 750 μmol m^–2^ s^–1^, respectively) and the lowest weights at 32°C (8.3; 11.2 and 17.9 g plant^–1^ at 200, 400 and 750 μmol m^–2^ s^–1^, respectively). The effect of air temperature on TDW at the final harvest was non-significant at 200 μmol m^–2^ s^–1^. At 400 and at 750 μmol m^–2^ s^–1^, however, the effect of air temperature on the dry matter production became increasingly significant for nearly all temperature combinations. The interaction between air and root-zone temperature was slightly significant (*P* = 0.036) and the plants grown at 20°C root zone temperature produced the lowest final dry weight, in all cases.

When the market weight was reached, shoot dry matter was 2.6, 3.8, and 4.2% at 200, 400, and 750 μmol m^–2^ s^–1^, respectively.

### Shoot Mass Fraction

**FIGURE 6:**
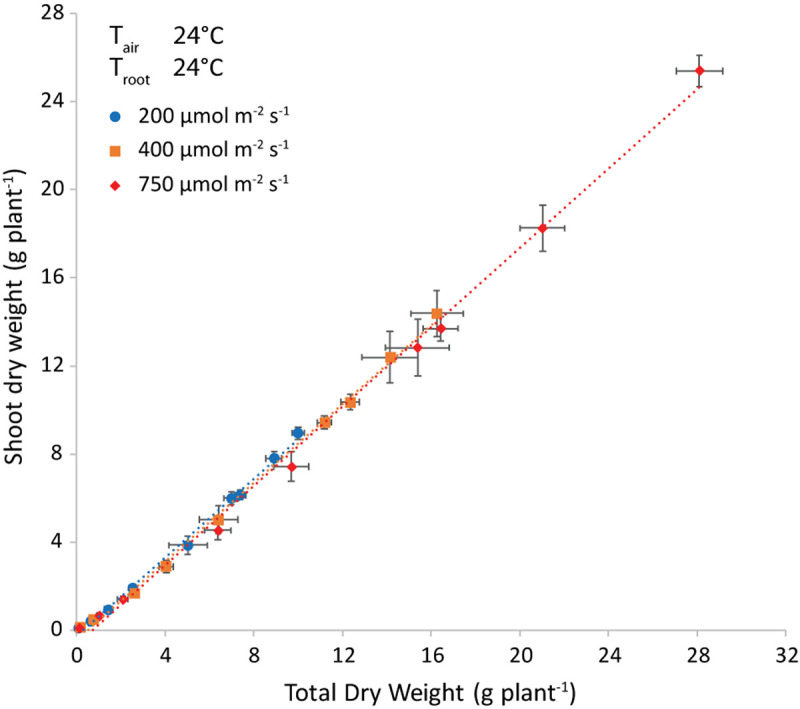
Shoot dry weight (g plant^–1^) plotted versus the total plant dry weight (g plant^–1^) and the corresponding trendlines at both air and root-zone temperatures of 24°C. Each symbol represents a different light intensity (200, 400 or 750 μmol m^–2^ s^–1^). The points are the average of four plants shown with the standard error of the mean.

Light intensity did not influence the allocation of dry matter to the shoot, as shown at air and root zone temperature of 24°C (Figure 6). Note that the best-fit lines were not forced through the origin, to account for preferential allocation to roots in the very early stages. The intercept with the *x*-axis is an estimate of the total weight at the end of this phase.

**FIGURE 7:**
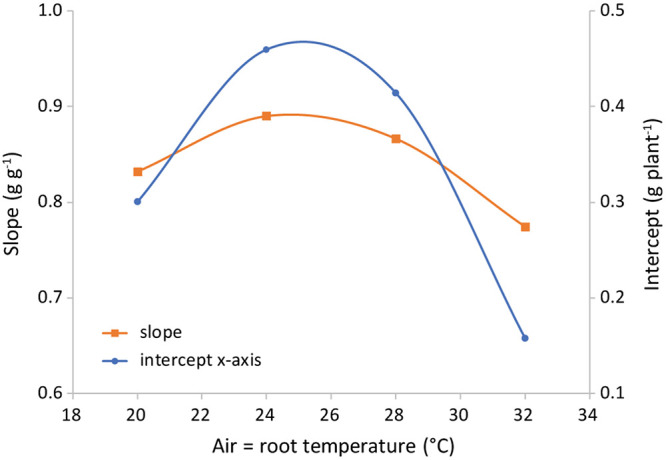
Representation of the parameters of the best-fit lines shown in Figure 6. Light intensities have been combined and root = air temperature. The slope is the shoot mass fraction (left axis) and the intercept with the *x*-axis (right axis) is an indication of the total plant dry weight at the end of the phase of preferential allocation to the roots.

The difference in regression lines under different light intensities was minimal for each combination of equal air and root zone temperature (Figure 6 and Supplementary Table 1). A single equation was fitted that combined the plants grown under different intensities. Both the effect of root temperature (examined at *T*_air_ = 24°C) and of air temperature (at *T*_root_ = 28°C) were minimal, but statistically significant (not shown). The joint effect was also minimal, yet visible at T_root_ = *T*_air_ (Figure 7 and Supplementary Figure 2) and statistically significant (*P* = 0.028).

### Specific Leaf Area

**FIGURE 8:**
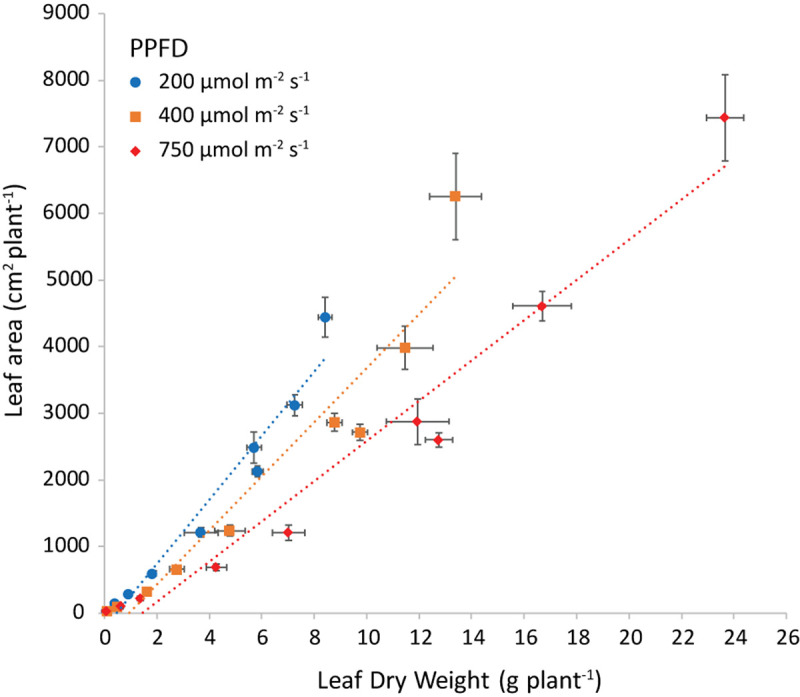
Leaf area (cm^2^ plant^−1^) plotted versus the leaf dry weight (g plant^−1^) at both air and root zone temperatures of 24°C and the three light intensities as indicated. The dotted lines represent the linear regression equations: at 200 μmol m^–2^ s^–1^ *y* = 444x (*R*^2^ = 0.9487), at 400 μmol m^–2^ s^–1^ *y* = 367x (*R*^2^ = 0.9112) and at 750 μmol m^–2^ s^–1^ *y* = 274x (*R*^2^ = 0.9474). The points are the average of four plants shown with the standard error of the mean.

**FIGURE 9:**
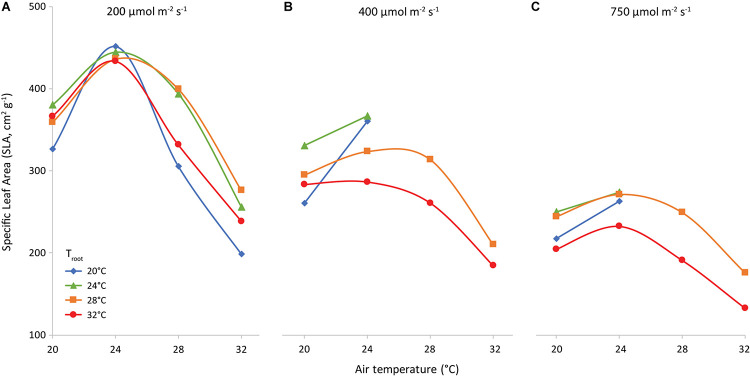
Specific Leaf Area (cm^2^ g^−1^) as affected by air temperature (*x*-axis) and root temperature (colors/symbols). Light intensities are 200 **(A)**, 400 **(B)** and 750 **(C)** μmol m^–2^ s^–1^, as indicated.

The slope of the regression line of leaf area against leaf dry weight (Figure 8) is the specific leaf area (SLA, cm^2^ g^–1^). Increasing light intensity notably reduced SLA. This trend was the same for all investigated combinations of air and root-zone temperature. Temperatures had a minor effect on SLA, where air temperature had a greater effect than root-zone temperature (Figure 9). At all light intensities the highest SLA was obtained at an air temperature of 24°C. A root-zone temperature of 28°C generally resulted in the highest SLA and 32°C in the lowest, but effects were minor. An increase in light intensity decreased SLA and reduced the influence of air temperature on SLA.

### Fresh Weight vs Dry Weight

The ratio of fresh to dry weight (the slope of the linear relationship between leaf fresh weight and leaf dry weight) showed a high *R*^2^ (>0.95) for any combination of PPFD, *T*_air_ and *T*_root_ (Supplementary Table 2). The lines were not forced through the origin, to account for the higher dry matter content of young plants.

**FIGURE 10:**
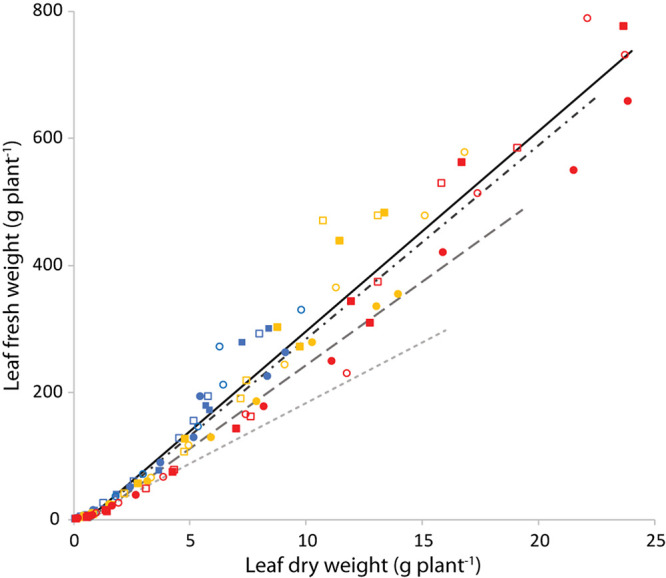
Leaf fresh weight (g plant^−1^) as a function of leaf dry weight (g plant^−1^) at an air temperature of 24°C. Blue is light intensity of 200 μmol m^–2^ s^–1^, yellow 400 and red 750 μmol m^–2^ s^–1^. The symbols indicate the root zone temperature, as follows: □ = 20°C; ■ = 24°C; ○ = 28°C and 🌑 = 32°C. The continuous line is the best fit of the points displayed (T_air_ = 24°C). The dashed lines are the best fit calculated for similar plots (see Supplementary Figure 4) with a T_air_ of 20°C (dash-dot), 28°C (long dash) and 32°C (short dash).

**FIGURE 11:**
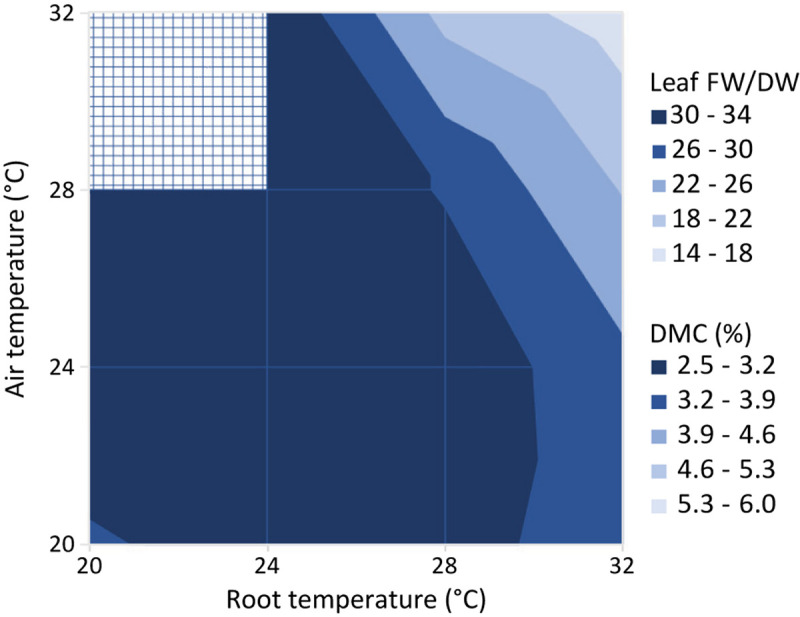
Ratio of leaf fresh to dry weight (Leaf FW/DW) and corresponding dry matter content (DMC, the ratio of leaf dry mass to leaf fresh mass in %). Values represent the slope of the regression line between the leaf fresh weight and leaf dry weight for all possible combinations of air and root zone temperature, while pooling light intensities together.

Figure 10 shows the leaf fresh weight vs leaf dry weight at an air temperature of 24°C, for all light intensities and root zone temperatures. Supplementary Figure 3 shows the fresh weight results of the four combinations with equal air and root zone temperature at all PPFD’s. Supplementary Figure 4 complements Figure 10 with the remaining air temperatures. Light intensity had little effect on leaf dry matter content. Figure 11 illustrates the slopes calculated by pooling light intensities together at a given combination of air and root zone temperature, as well as the corresponding leaf dry matter content.

### Light Use Efficiency

**FIGURE 12:**
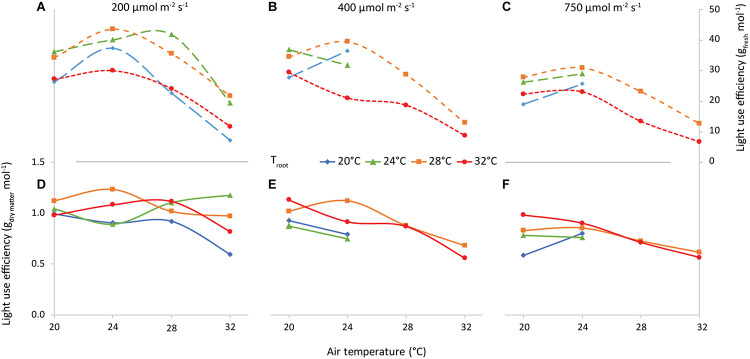
Light Use Efficiency vs air temperature, for light intensities of 200 **(A,D)**, 400 **(B,E)** and 750 **(C,F)** μmol m^–2^ s^–1^, as indicated. The colors/symbols represent the root-zone temperatures. The top graphs **(A–C)** illustrate shoot fresh weight and the bottom graphs **(D–F)** illustrate shoot dry weight production per unit of incident light (g mol^–1^).

A main factor determining the feasibility of vertical farming is the ratio “fresh produce (g m^–2^) per unit of incident light (mol m^–2^)” (LUE_FW_, Figure 12). Averaged over all air and root-zone temperature combinations, the LUE_FW_ was 29, 27, and 21 g FW per mol incident light at 200, 400, and 750 μmol m^–2^ s^–1^, respectively. LUE_FW_ was highest at 24°C air temperature and lowest at 32°C for all 3 light intensities. Root-zone temperature also had a clear effect, where 28°C generally resulted in the highest LUE_FW_ and 32°C the lowest.

The combination of air and root-zone temperature had little to no effect on the dry matter per unit of incident light (LUE_DW_). The effect of increasing light intensity on LUE_DW_ was about as large as on LUE_FW_ (1.01, 0.87, and 0.76 g DW per mol incident light at 200, 400, and 750 μmol m^–2^ s^–1^, respectively).

## Discussion

This study was aimed at providing quantitative information on lettuce crop growth that is relevant for good climate management in plant factories. The final aim was to explain yield response to light intensity (other than assimilation), air temperature and root zone temperature, which was mediated through the effect of these variables on biomass allocation, SLA and fresh weight accumulation (Figure 1). The high incidence of tip burn in the treatments that combined the two highest air temperatures with the two lowest root temperatures at the middle and high light intensity did not allow us to explore all planned combinations of these variables (8 out of 48 were excluded). Nevertheless, we believe that our results provided useful information for climate management in plant factories and advanced our knowledge about relevant processes, particularly the accumulation of fresh weight.

### Biomass Production and Light Use Efficiency

The light use efficiency of shoot dry matter production (LUE_DW_) was 1.01, 0.87, and 0.76 g mol^–1^ incident light at 200, 400, and 750 μmol m^–2^ s^–1^, respectively, when averaged over all temperature combinations. A decrease in LUE_DW_ at increased light intensity was also observed by Fu et al. (2012) for 200 up to 800 μmol m^–2^ s^–1^. This decrease can be explained by the saturation-type photosynthesis-light response curve. This also explains why Kelly et al. (2020) did not find a decrease in LUE_DW_ as their PPFD levels were much lower (120 to 270 μmol m^–2^ s^–1^ at 16 h daylength) than in our experiment. Here the highest LUE was achieved at 200 μmol m^–2^ s^–1^, 24°C air temperature and 28°C root temperature: 44 g FW and 1.23 g DW per mol incident light. This was approximately 30% higher than Pennisi et al. (2020), who observed a LUE of about 0.9 g DW per mol incident light at 200 μmol m^–2^ s^–1^. Although they had a shorter crop cycle and an exclusively red-blue spectrum, we reason that the difference in CO_2_ concentration is the most likely cause: Pennisi et al. (2020) grew lettuce at 450 μmol mol^–1^ CO_2_, whereas we used 1,200 μmol mol^–1^. An increase in LUE of 30% as a result of this difference is plausible (Nederhoff, 1994). Zou et al. (2019) reported an even lower LUE of only 0.6 g DW per mol incident light at 200 μmol m^–2^ s^–1^ and ambient CO_2_.

The yield in the experiments of Pennisi et al. (2020) did not increase for light intensities exceeding 250 μmol m^–2^ s^–1^, which indicates that CO_2_ concentration may have been the limiting factor. Fu et al. (2012) also observed no difference is shoot weight between PPFD 400 and 600 μmol m^–2^ s^–1^ and a lower shoot weight at 800 μmol m^–2^ s^–1^ for lettuce grown at 400 μmol mol^–1^ CO_2_. Indeed, Pérez-López et al. (2013) observed that an individual or combined increase in light intensity (from 400 to 700 μmol m^–2^ s^–1^) and CO_2_ concentration (from 400 to 700 μmol mol^–1^) could significantly increase yield of a Batavia variety of lettuce (up to 77%). Duggan-Jones and Nichols (2014) did not observe saturation with light up to 480 μmol m^–2^ s^–1^ in lettuce at 1,000 μmol mol^–1^ CO_2_. Frantz et al. (2004) observed an increase of dry matter production even up to their maximum PPFD of 1,000 μmol m^–2^ s^–1^, with a CO_2_ concentration of 1,200 μmol mol^–1^.

In our experiment total dry mass increased with light intensity up to our highest intensity (750 μmol m^–2^ s^–1^), although the relative increase was lower at higher light intensities. It was of course rather unfortunate that the light spectrum was not identical at all intensities (section “Growth conditions, treatments and analysis” and Supplementary Figure 1). Nevertheless, as the spectrum was the same for the two highest light intensities, we can safely conclude that light was the limiting factor in well-managed lettuce production, even at a high light intensity of 750 μmol m^–2^ s^–1^ (DLI of 43.2 mol m^–2^ d^–1^).

### Shoot Mass Fraction and Specific Leaf Area

An important parameter in understanding the amount of fresh weight produced per mol of incident light is the fraction of light intercepted, which depends on the leaf area index. The formation of thin leaves (high SLA) results in more leaf area for the same leaf dry mass and hence a quicker build-up of light interception and higher plant growth rate [e.g., shown by Heuvelink (1989) for young tomato plants]. Furthermore, the partitioning of a high proportion of assimilates toward the leaves is also important to quickly build up leaf area index in the early stages of crop growth. In our case, SMF was not influenced by light intensity (Figure 6), whereas we would expect a higher SMF at low light intensity according to the functional equilibrium (Poorter and Nagel, 2000). However, in their meta-analyses, Poorter et al. (2012, 2019), reported a minimal effect on mass allocation of Daily Light Integrals above approximately 10 mol m^–2^ d^–1^. As the lowest light intensity in our experiment was equivalent to 11.5 mol m^–2^ d^–1^, this might explain the absence of a light intensity effect on SMF.

Increasing light interception may be attained more efficiently at no cost to the root system, by making “thinner” leaves and consequently increasing SLA. In our experiment, SLA was lower at higher light intensity (Figures 8, 9). This is a well-known response (meta-analysis by Evans and Poorter, 2001) and was also shown by Kitaya et al. (1998) in lettuce grown in a growth chamber. A decrease in SLA negates the positive effect of light intensity on total dry matter production. Light intensity did not have an effect on leaf area for up to 15 days after transplanting as a result of the adaptation in SLA (Supplementary Figure 5). The effect of temperature on SLA (Figure 9) is less documented, although Rosbakh et al. (2015) revealed a very weak positive SLA-temperature correlation. However, there is evidence that SLA across species correlates with the temperature of their habitat (Atkin et al., 2006). Low rates of cell expansion at low temperatures may lead to a large number of small cells per unit area, resulting in smaller and denser leaves on plants in cold habitats (Poorter et al., 2009). By transplanting plants of the same genotype at three different heights in the Bavarian Alps, Scheepens et al. (2010) demonstrated that temperature can cause intraspecific variation in SLA. Frantz et al. (2004) showed a strong influence of air temperature on leaf expansion in lettuce grown in a growth chamber (600 μmol m^–2^ s^–1^; 34.6 mol m^–2^ d^–1^; and 1,200 μmol mol^–1^CO_2_ concentration), the highest expansion rate being at 27 and 30°C, and the lowest at 33°C. Even though expansion rate is not exactly SLA, one can conclude that literature corroborates the observed trend of the SLA temperature relationship. The temperature at which the maximum SLA is attained differs from literature and might well depend on cultivar.

### Leaf Fresh to Dry Weight Ratio

The only variable that affected the ratio of leaf fresh weight to dry weight was air temperature (Figure 10). The observed trend with a maximum at 24°C was similar to the trend of SLA with temperature (Figure 9). The correlation between temperature and cell size of lettuce has been known since Bensink (1971) demonstrated that an increase in temperature from 10 to 30°C increased average cell diameter by 68% without an effect on cell number. Conversely, light intensity did increase cell number while decreasing cell size. Bensink (1971) concluded that “growth increments are entirely due to a proportional increase in cell size,” which is either caused by or correlated with temperature. This explains the similarity of the temperature trend of SLA with the trend of water content (Figure 10), assuming that cell dry matter does not increase proportionally with size. As the response of SMF to temperature (Figure 7) is very similar, there seems to be a correlation between sink strength and leaf expansion. Altogether, the temperature effect on these partial processes of leaf area development and mass allocation explains the (small) temperature effect observed on total dry matter production.

### Climate Management

Our results confirmed the optimal day-time temperature for lettuce production of 24°C (Marsh and Albright, 1991; Thompson et al., 1998). In spite of the small decrease of yield observed at 28°C, the 30°C optimal temperature proposed by Frantz et al. (2004) is certainly beyond the limit (of this cultivar).

Even though root-zone temperature had a limited effect on the dry weight production, it had some effect on the water-related processes (Figure 11), and ultimately on the light use efficiency of fresh weight (Figure 12). The fact that the occurrence of tip burn was highest at 32°C (*T*_air_ = *T*_root_, not shown) would be caution enough against high root zone temperatures. He and Lee (1998) found no direct effect of root zone temperature (15–25°C) in all indicators of growth of three lettuce cultivars, either shaded or unshaded, but growth was much reduced when there was no root zone temperature control, in the tropical conditions of Singapore. Furthermore, we certainly cannot state that optimal root temperature depends on light intensity [as Gosselin and Trudel (1984) observed with tomato] since *T*_root_ = 28°C seems optimal for nearly all performance indicators at all light intensities. Nevertheless, in the hydroponic growing systems typical of lettuce in plant factories, a most reasonable compromise would be *T*_root_ = *T*_air_ = 24°C, which disposes of the need for heating the nutrient solution.

The yield per mol of incident light was determined by several plant parameters (Figure 12). The ratio between fresh and dry shoot weight was not influenced by light intensity nor root zone temperature, but was reduced at higher air temperatures (Figure 10). Therefore an air temperature not exceeding 24°C seems to warrant the highest amount of water for a given quantity of dry matter in the leaves. On the other hand, the lack of an effect of PPFD on leaf area (until about 15 days after transplanting) implied that the decrease in SLA perfectly balanced the increase in shoot dry matter (see Figure 1). To this end, the positive feedback of dry matter production and light interception is broken and the fraction of light that is intercepted by young plants is independent of light intensity.

## Conclusion

This study was aimed at providing quantitative information that is relevant for good climate management of lettuce crops in plant factories. In particular, we have analyzed the relationship between light intensity, air temperature and root-zone temperature and lettuce growth, at non-limiting CO_2_ concentration.

When other factors are not limiting, dry weight production increases with increasing light intensity until the maximum investigated PPFD of 750 μmol m^–2^ s^–1^. Nevertheless, as the efficiency of light use for both dry and fresh weight production decreased with increasing light intensity, the optimal light intensity has to be determined in view of the value of the crop and the capital and running cost of light. Fresh and dry yield, SLA, shoot mass allocation and water content of leaves showed the same trend with air temperature, with a maximum around 24°C. On the other hand, the effect of root temperature was less prominent, with an optimum around 28°C in nearly all conditions.

## Data Availability Statement

The dataset collected during the experiments is available at https://doi.org/10.4121/5a7472f0-8a67-4ec0-84c9-9d032990d654.

## Author Contributions

The experiment was designed by LG (who obtained the funding) and EM. EM prepared the protocol for the measurements. FP and LC executed the experiments, with the help and supervision of MB and LG. LC and FP performed the first analysis of the results and LC drafted the manuscript. EH and CS supervised the analysis and wrote the discussion. The final version of the manuscript has been reviewed and completed by each author. All authors contributed to the article and approved the submitted version.

## Conflict of Interest

The authors declare that the research was conducted in the absence of any commercial or financial relationships that could be construed as a potential conflict of interest.

## Footnotes

1 Corrections were not required during any of the treatments.

## Supplementary Material

Supplementary Figure 1 Spectral distribution of the two types of lamps used. The spectrum of the two highest intensities was measured separately and found to be identical. The value in the *y*-axis is normalized so that $\int_{\lambda =400}^{\lambda =800}$ measured intensity dλ = 1.

Supplementary Figure 2 Shoot dry weight (g plant^–1^) plotted vs total plant dry weight (g plant^–1^) at equal air and root zone temperature. Each color represents all light intensities at a different temperature combination. *R*^2^ was in all cases above 0.995. Regression lines were not forced through the origin and all are statically different at 99% confidence interval, except for the ones at 24/24°C and 28/28°C that are different at 95% confidence interval.

Supplementary Figure 3 Leaf fresh weight (g plant^–1^) as a function of leaf dry weight (g plant^–1^) for the combinations with same air temperature and root zone temperature, pooled for light intensity. Each trendline represents a different temperature combination: 20/20°C *y* = 0.0358x (*R*^2^ = 0.9622), 24/24°C *y* = 0.0308x (*R*^2^ = 0.9555), 28/28°C *y* = 0.035 8x (*R*^2^ = 0.9622), 32/32°C *y* = 0.0643x (*R*^2^ = 0.9822).

Supplementary Figure 4 Leaf fresh weight (g plant^–1^) as a function of leaf dry weight (g plant^–1^) at air temperatures 20 **(A)**, 28 **(B)** and 32°C **(C)**, as indicated. Blue is light intensity of 200, yellow 400 and red 750 μmol m^–2^ s^–1^. The symbols indicate the root zone temperature, as follows: □ = 20°C; ■ = 24°C; ○ = 28° and 🌑 = 32°C. The full line is the best fit of all points displayed, and is reported in Figure 9 in the main text.

Supplementary Figure 5 Evolution in time (Days after Transplanting) of Leaf area (cm^2^ plant^–1^), for the “best” temperature combination (T_air_ 24 and T_root_ 28°C), at the three light intensities, as indicated.

Supplementary Table 1 Linear regression equations for SDW vs TDW at the same air and root zone temperature, for the different light intensities. The intercept with the *x*-axis is an indication of the total dry weight at the end of the phase with preferential allocation to roots. Within the same temperature the regression lines do not statically differ at 99% confidence interval.

Supplementary Table 2 Linear equations for leaf fresh weight vs leaf dry weight at different light intensities at equal air and root zone temperatures.
