# Correction: Plant factories are heating up: hunting for the best combination of light intensity, air temperature and root-zone temperature in lettuce production

**DOI:** 10.3389/fpls.2025.1744137

**Published:** 2025-12-18

**Authors:** Laura Carotti, Luuk Graamans, Federico Puksic, Michele Butturini, Esther Meinen, Ep Heuvelink, Cecilia Stanghellini

**Affiliations:** 1 Department of Biological, Geological and Environmental Sciences, Alma Mater Studiorum, University of Bologna, Bologna, Italy; 2 Greenhouse Horticulture, Wageningen University and Research, Wageningen, Netherlands; 3 Horticulture and Product Physiology, Wageningen University and Research, Wageningen, Netherlands

**Keywords:** climate management, dry matter allocation, efficiency, leaf expansion, production climate, resource use efficiency, vertical farm, light use efficiency

The **Data availability statement** was erroneously given as “The raw data supporting the conclusions of this article will be made available by the authors, without undue reservation”.

The correct **Data availability statement** is “The dataset collected during the experiments is available at https://doi.org/10.4121/5a7472f0-8a67-4ec0-84c9-9d032990d654“.

The original version of this article has been updated.

