# Supplementary material for: Plant Factories Are Heating Up: Hunting for the Best Combination of Light Intensity, Air Temperature and Root-Zone Temperature in Lettuce Production
Source: Front Plant Sci. 2021 Jan 28;11:592171. doi: 10.3389/fpls.2020.592171 (PMC7876451; doi:10.3389/fpls.2020.592171)
Supplement: Supplementary Table 1 — Linear regression equations for SDW vs TDW at the same air and root zone temperature, for the different light intensities. The intercept with the x-axis is an indication of the total dry weight at the end of the phase with preferential allocation to roots. Within the same temperature the regression lines do not statically differ at 99% confidence interval. [file Table_1.docx]

***Supplementary Table 1.*** *Linear regression equations for SDW vs TDW at the same air and root zone temperature, for the different light intensities. The intercept with the x-axis is an indication of the total dry weight at the end of the phase with preferential allocation to roots. Within the same temperature the regression lines do not statically differ at 99% confidence interval.*

| **Air=root zone temperature (°C)** | **Light intensity (*µmol m^-2^s^-1^****)* | **Equation** | **Intercept x-axis (g/plant)** |
| --- | --- | --- | --- |
| 20 | 200 | y=0.8215x-0.1611 (R^2^=0.9981) | 0.20 |
|  | 400 | y=0.8372x-0.2871 (R^2^=0.9983) | 0.34 |
|  | 750 | y=0.8354x-0.3216x (R^2^=0.9962) | 0.38 |
| 24 | 200 | y=0.8595x-0.2765 (R^2^=0.9961) | 0.32 |
|  | 400 | y=0.8391x-0.4402 (R^2^=0.9974) | 0.52 |
|  | 750 | y=0.8996x-0.6626 (R^2^=0.9964) | 0.74 |
| 28 | 200 | y=0.8701x-0.2335 (R^2^=0.9969) | 0.27 |
|  | 400 | y=0.8658x-0.3879 (R^2^=0.9957) | 0.45 |
|  | 750 | y=0.8755-0.5587x (R^2^=0.9966) | 0.64 |
| 32 | 200 | y=0.7523x-0.0323 (R^2^=0.9982) | 0.04 |
|  | 400 | y=0.7757x-0.1255 (R^2^=0.9983) | 0.16 |
|  | 750 | y=0.7797x-0.1744 (R^2^=0.9986) | 0.22 |
