# Supplementary material for: Plant Factories Are Heating Up: Hunting for the Best Combination of Light Intensity, Air Temperature and Root-Zone Temperature in Lettuce Production
Source: Front Plant Sci. 2021 Jan 28;11:592171. doi: 10.3389/fpls.2020.592171 (PMC7876451; doi:10.3389/fpls.2020.592171)
Supplement: Supplementary Table 2 — Linear equations for leaf fresh weight vs leaf dry weight at different light intensities at equal air and root zone temperatures. [file Table_2.docx]

***Supplementary Table 2.*** *Linear equations for leaf fresh weight vs leaf dry weight at different light intensities at equal air and root zone temperatures.*

| **Air = root zone temperature (°C)** | **Light intensity**  **(*µmol m^-2^s^-1^****)* | **Equation** |
| --- | --- | --- |
| 20/20 | 200 | y = 28.570x-8.1306 (R^2^ = 0.9936) |
|  | 400 | y = 30.432x-20.115 (R^2^ = 0.9886) |
|  | 750 | y = 29.807x-29.493 (R^2^ = 0.9689) |
| 24/24 | 200 | y = 36.853x-21.216 (R^2^ = 0.9660) |
|  | 400 | y = 37.137x-29.650 (R^2^ = 0.9737) |
|  | 750 | y = 33.383x-43.174 (R^2^ = 0.9765) |
| 28/28 | 200 | y = 32.165x-16.877 (R^2^ = 0.9766) |
|  | 400 | y = 30.400x-27.067 (R^2^ = 0.9814) |
|  | 750 | y = 30.294x-45.951 (R^2^ = 0.9622) |
| 32/32 | 200 | y = 17.944x-3.4638 (R^2^ = 0.9983) |
|  | 400 | y = 16.844x-7.2501 (R^2^ = 0.9862) |
|  | 750 | y = 15.798x-7.1954 (R^2^ = 0.9945) |
